# Horsefly reactions to black surfaces: attractiveness to male and female tabanids versus surface tilt angle and temperature

**DOI:** 10.1007/s00436-020-06702-7

**Published:** 2020-05-19

**Authors:** Gábor Horváth, Ádám Pereszlényi, Ádám Egri, Benjamin Fritz, Markus Guttmann, Uli Lemmer, Guillaume Gomard, György Kriska

**Affiliations:** 1 Environmental Optics Laboratory, Department of Biological Physics, ELTE Eötvös Loránd University, Pázmány sétány 1, Budapest, H-1117 Hungary; 2 Department of Zoology, Hungarian Natural History Museum, Ludovika tér 2-6, Budapest, H-1083 Hungary; 3 MTA Centre for Ecological Research, Danube Research Institute, Karolina út 29-31, Budapest, H-1113 Hungary; 4 MTA Centre for Ecological Research, Evolutionary Systems Research Group, Klebelsberg Kuno utca 3, Tihany, H-8237 Hungary; 5 Light Technology Institute, Karlsruhe Institute of Technology (KIT), Engesserstrasse 13, D-76131 Karlsruhe, Germany; 6 Institute of Microstructure Technology, Karlsruhe Institute of Technology (KIT), Eggenstein-Leopoldshafen, D-76344 Karlsruhe, Germany; 7 Group for Methodology in Biology Teaching, Biological Institute, ELTE Eötvös Loránd University, Pázmány sétány 1, Budapest, H-1117 Hungary

**Keywords:** Tabanid fly, Horsefly, Sticky insect trap, Tilted surface, Polarised light, Polarotaxis, Polarization vision, Thermoreception, Behavioural ecology

## Abstract

Tabanid flies (Diptera: Tabanidae) are attracted to shiny black targets, prefer warmer hosts against colder ones and generally attack them in sunshine. Horizontally polarised light reflected from surfaces means water for water-seeking male and female tabanids. A shiny black target above the ground, reflecting light with high degrees and various directions of linear polarisation is recognised as a host animal by female tabanids seeking for blood. Since the body of host animals has differently oriented surface parts, the following question arises: How does the attractiveness of a tilted shiny black surface to male and female tabanids depend on the tilt angle δ? Another question relates to the reaction of horseflies to horizontal black test surfaces with respect to their surface temperature. Solar panels, for example, can induce horizontally polarised light and can reach temperatures above 55 °C. How long times would horseflies stay on such hot solar panels? The answer of these questions is important not only in tabanid control, but also in the reduction of polarised light pollution caused by solar panels. To study these questions, we performed field experiments in Hungary in the summer of 2019 with horseflies and black sticky and dry test surfaces. We found that the total number of trapped (male and female) tabanids is highest if the surface is horizontal (δ = 0°), and it is minimal at δ = 75°. The number of trapped males decreases monotonously to zero with increasing δ, while the female catch has a primary maximum and minimum at δ = 0° and δ = 75°, respectively, and a further secondary peak at δ = 90°. Both sexes are strongly attracted to nearly horizontal (0° ≤ δ ≤ 15°) surfaces, and the vertical surface is also very attractive but only for females. The numbers of touchdowns and landings of tabanids are practically independent of the surface temperature *T*. The time period of tabanids spent on the shiny black horizontal surface decreases with increasing *T* so that above 58 °C tabanids spent no longer than 1 s on the surface. The horizontally polarised light reflected from solar panels attracts aquatic insects. This attraction is adverse, if the lured insects lay their eggs onto the black surface and/or cannot escape from the polarised signal and perish due to dehydration. Using polarotactic horseflies as indicator insects in our field experiment, we determined the magnitude of polarised light pollution (being proportional to the visual attractiveness to tabanids) of smooth black oblique surfaces as functions of δ and *T*.

**Electronic supplementary material:**

The online version of this article (10.1007/s00436-020-06702-7) contains supplementary material, which is available to authorized users.

## Introduction

Tabanid flies (Diptera: Tabanidae) are visually attracted to shiny black targets (Bracken et al. 1962; Thorsteinson et al. 1966; Granger 1970; Roberts 1970; Browne and Bennett 1980; Allan and Stoffolano 1986; Sasaki 2001; Lehane 2005; Mihok and Mulye 2010; Krcmar 2013; Baldacchino et al. 2014; Horváth et al. 2014a). They are polarisation-sensitive and positively polarotactic (Egri et al. 2012a). Horizontally polarised light reflected from surfaces on the ground means water for water-seeking male and female tabanids (Horváth et al. 2008; Kriska et al. 2009). If the shiny black target lies above the ground and reflects light with high degrees and various directions of linear polarisation, the optical signal means host animal for female tabanids seeking a blood meal (Egri et al. 2012a; Horváth et al. 2017). Based on these two different kinds of polarotaxis in tabanids, efficient L-shaped polarisation horsefly traps have been developed (Horváth et al. 2014b). One of these polarising traps consists of a horizontal and a vertical surface, both being shiny, black and sticky (Egri et al. 2013): the horizontal component traps water-seeking male and female tabanids, while the vertical element exclusively captures host-seeking females. It is an interesting visual ecological question how the female per male ratio changes with the tilt angle when the orientation of the trap surface varies from horizontal to vertical. Since the body of host animals has differently oriented (horizontal, oblique, vertical) surface parts (Horváth et al. 2010a), the same question arises: How does the attractiveness to male and female tabanids of a tilted surface depend on its tilt angle?

The answer of this question is important not only in tabanid control, but also in the reduction of polarised light pollution caused by solar panels. It is well known that shiny black solar panels attract many different polarotactic insects, especially aquatic insects and insects associated with water (Horváth et al. 2010b; Blahó et al. 2012a; Száz et al. 2016). These insects detect water by means of the horizontal polarisation of water-reflected light (Horváth and Csabai 2014), and thus are attracted to all smooth black objects reflecting horizontally polarised light (Horváth and Kriska 2008). This visual attraction is adverse if the lured insects either lay their eggs onto the black surface or cannot escape from the polarised signal and perish due to dehydration. This phenomenon is called polarised light pollution (Horváth et al. 2009, 2014c). Solar panels are usually tilted and oriented toward south (on the northern hemisphere of the Earth) to maximize the energy yield. The optimal tilt angle depends on the geographical latitude. In Hungary (at 47° northern latitude), for example, 30° from the horizontal is the optimal tilt angle of solar panels (Jacobson and Jadhav 2018). How does the polarised light pollution of solar panels depend on the tilt angle? To answer the above two questions, we performed a field experiment in Hungary in the summer of 2019 with horseflies and oblique sticky shiny black test surfaces.

Blood-sucking horseflies prefer warmer (sunlit, darker) host animals against colder (shady and/or brighter) ones and generally attack them in sunshine (Tashiro and Schwardt 1953; Bracken and Thorsteinson 1965; Horváth et al. 2010a, 2019; Blahó et al. 2012b, 2013; Egri et al. 2012b; Krcmar et al. 2014). But if a surface is too hot for a horsefly, it may leave the surface promptly after landing and search for a more appropriate host. If the surface of a solar panel is too hot, this can reduce the negative effect of polarised light pollution. In practice, the operating temperature of a solar cell in outdoor conditions is typically 50–55 °C or higher (Sato and Yamada 2019). So in outdoor conditions, it is likely that horseflies will not spend a lot of time over solar panels. How does the reaction of horseflies to matte (rough) and shiny (smooth) horizontal solar panels depend on the surface temperature? Another field experiment was designed to answer this question.

Hence, using polarotactic horseflies as indicator species, in our two field experiments we determined the visual attractiveness to polarotactic insects of smooth black oblique test surfaces as functions of both tilt angle and surface temperature. Here, we present the results of these two field tests.

## Materials and methods

### Field experiment 1: influence of the surface tilt angle

The reflection-polarisation characteristics of our test surfaces used in field experiment 1 were measured by imaging polarimetry (Horváth and Varjú 2004) in the red (650 nm), green (550 nm) and blue (450 nm) parts of the spectrum. Here, we present only the polarisation patterns measured in the blue (450 nm) part of the spectrum, because practically the same patterns were obtained in the red and green spectral ranges.

Field experiment 1 was performed between June 30 and August 30, 2019, on a Hungarian horse farm near Szokolya (47° 52′ North, 19° 00′ East), where tabanid flies were in abundance. To study the influence of the tilt angle δ of shiny black sticky test surfaces on the attractiveness to tabanids, nine wooden plates (50 cm × 50 cm) were used (Supplementary Fig. S1A). One side was painted black with a common oil paint, while the other side remained in its original matte bright beige colour. The tilt angles considered herein were δ = 0° (horizontal), 15°, 30°, 45°, 60°, 75°, 90° (vertical), 120° and 135°. The horizontal test surface was simply laid on the grassy ground. The test surfaces were set up 50 cm apart from each other along a straight line. Between sunrise and sunset, all test surfaces were exposed to direct sun- and skylight. Prior to sunset, the test surfaces were in the shadow of the near vegetation. The black side of the test surfaces was covered by a transparent, colourless, odourless and weatherproof insect-monitoring adhesive (BabolnaBio®, Hungary). We periodically removed and counted the tabanids trapped by the sticky test surfaces (Supplementary Fig. S1B-E). During this process, we determined the sex of trapped horseflies on the basis of the existence (female) or non-existence (male) of an ommatidia-free thin zone between the two compound eyes. The slight non-uniformity of the collecting periods was purposive: after cool, rainy and windy weather, the counting period was longer with a few days to compensate the decrease of tabanid flight activity. After that the surfaces were cleaned (with manual removing of all insects trapped), their order was randomized, and the adhesive was refreshed. Thus, the altered situation after each tabanid counting represented a new replication. In our experiment, the number of replications was 6 over a total duration of 62 days. These numbers were large enough to detect statistical differences in the numbers of trapped tabanids.

The species level identification of tabanids in experiment 1 was impossible since the specimens were seriously damaged. It was obvious, however, that they were tabanids (Diptera: Tabanidae). In previous field experiments (Herczeg et al. 2014, 2015), the following tabanid species were found to occur at the same study site: *Tabanus tergestinus*, *Tabanus bromius*, *Tabanus bovinus*, *Tabanus autumnalis*, *Atylotus fulvus*, *Atylotus loewianus*, *Atylotus rusticus*, *Haematopota italica*.

### Field experiment 2: influence of the surface temperature

The temperature of the horizontal test surfaces was measured with a contact thermometer (GAO Digital Multitester EM392B 06554H, EverFlourish Europe Gmbh., Friedrichsthal, Germany, nominal precision of ± 1 °C). In one case, thermograms of the matte and glass-covered matte test surfaces were measured with a thermocamera (VarioCAM®, Jenoptik Laser Optik Systeme GmbH, Jena, Germany, nominal precision of ± 1.5 °C).

In order to study the behaviour of tabanids on horizontal black test surfaces as a function of the surface temperature, we conducted field experiment 2 between June 18 and July 6, 2019, on five sunny and warm days at the same horse farm in Szokolya where experiment 1 was performed. We used two different black (rearside of the surfaces was painted black using three layers of acrylic colour) horizontal test surfaces (50 cm × 50 cm). *m*, matte black solar panels composed of 20 rectangular elements (12.5 cm × 10 cm) with 15 elementary cells (3.3 cm × 2.5 cm) and coated by a micro/nanotextured cover layer obtained by a bio-replication process of natural rose petals in polymethyl methacrylate (PMMA, see Hünig et al. 2016; Fritz et al. 2018, 2020a). *g* + *m*, an identical matte black solar panel mimicking a surface covered with a common glass pane of 3 mm thickness. The horizontal test surfaces were laid on the grassy ground along a straight line 50 cm apart from each other. Their order was randomized in every 30 min. They were exposed to direct sun- and skylight and were never in the shade of vegetation. An observer wearing white clothes counted the following reactions of tabanids from a chair placed at a distance of 2 m from the test surfaces: touchdown, landing, time period (in seconds) of staying after landing. In experiment 2, horseflies were not collected.

### Statistical analysis

The numbers of tabanids trapped by the differently tilted test surfaces were compared with factorial ANOVA with Tukey’s HSD post hoc test. Independent variables were the tilt angle δ and sex (male, female) of tabanids. The test was performed with the use of the software Statistica 7.0. Furthermore, we applied the Wilcoxon signed-rank test (R Statistics 3.2.3) to find differences in the attractiveness of the horizontal solar panels.

## Results

### Influence of surface tilt angle on tabanid attraction (experiment 1)

**Fig. 1:**
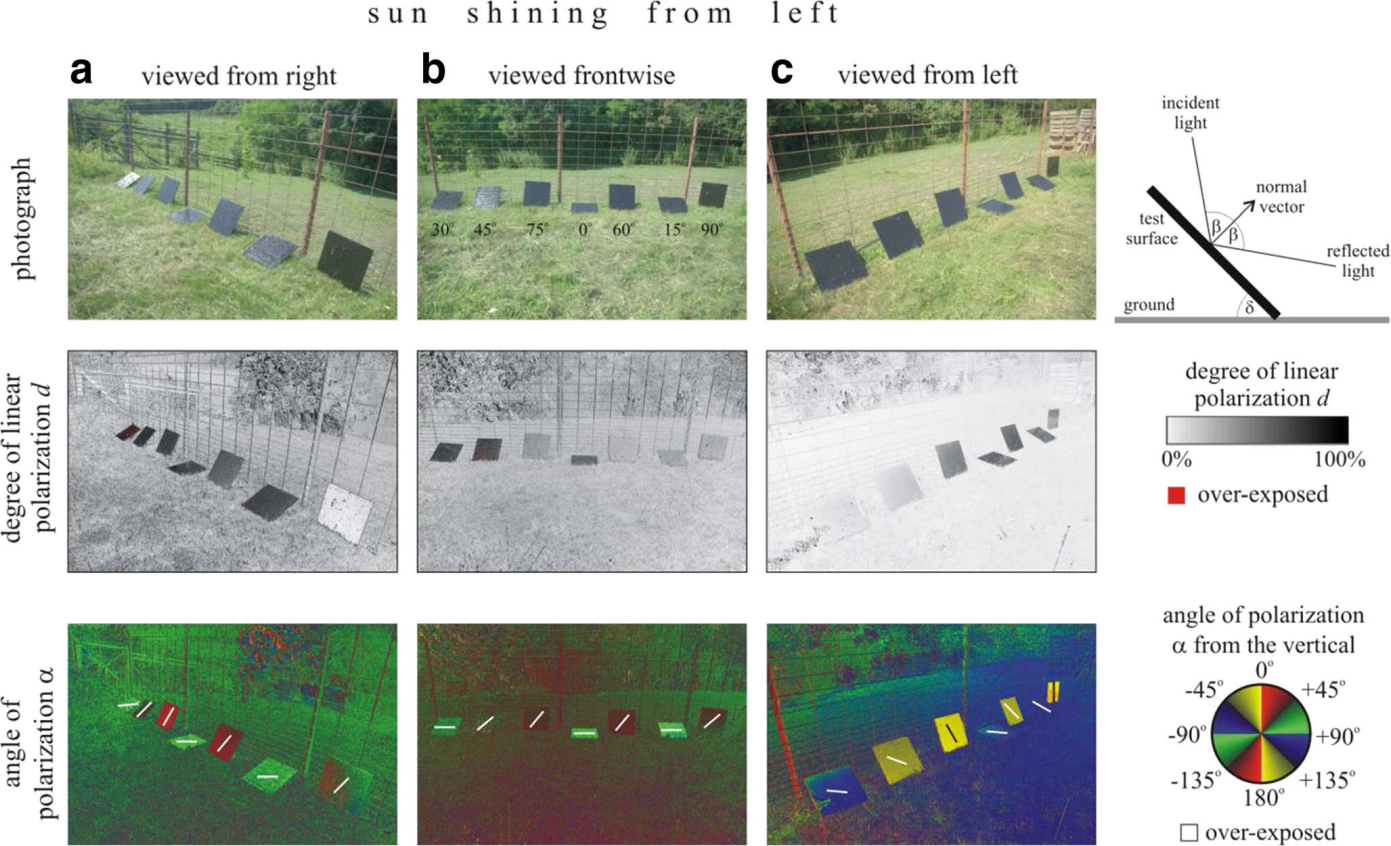
Photographs and patterns of the degree of linear polarisation *d* and of the angle of polarisation α (measured clockwise from the vertical) of the tilted shiny black sticky test surfaces used in field experiment 1 and measured with imaging polarimetry in the blue (450 nm) spectral range from three different viewing directions (from right, front, from left) relative to the straight line of the series of surfaces. The sun shone from the left side. The tilt angle of the optical axis of the polarimeter was − 30° from the horizontal. In the photo of column B, the tilt angles of the test surfaces are given. The (white, black) bars in the α-patterns represent the local directions of polarisation. Inset in the top right corner, geometry of light reflection from a tilted test surface. δ, tilt angle; β, angle of reflection

**Fig. 2:**
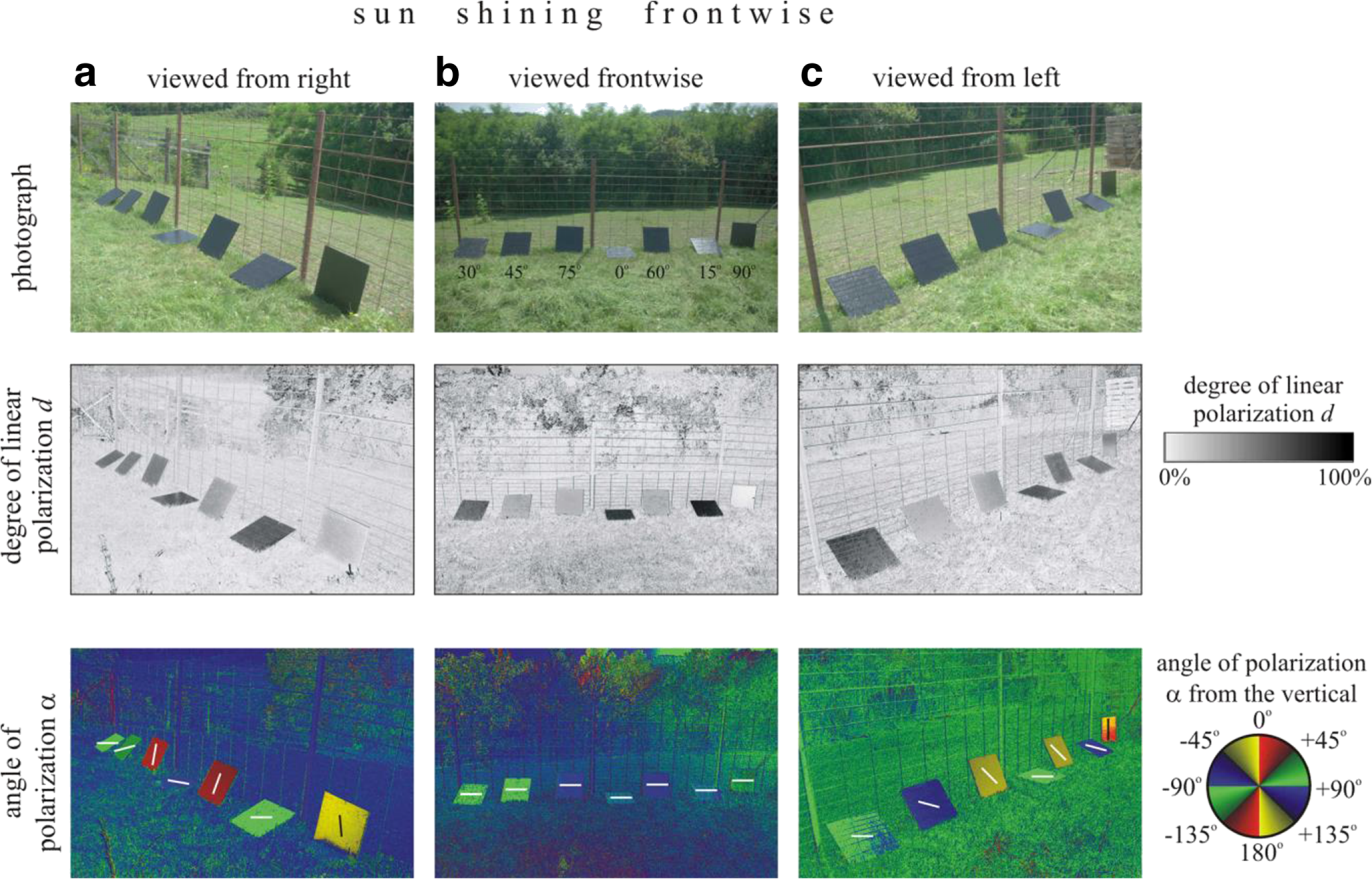
As in Fig. 1 but here, the sun shone front

Both degree *d* and angle α of linear polarisation of light reflected from a black test surface depend on the angle of reflection β determined by the tilt angle δ of the surface and the direction of view of the polarimeter (observer or flying tabanid fly) relative to the normal vector of the surface as seen in Figs. 1 and 2: *d* is maximal when light is reflected under the Brewster’s angle θ_B_ (= arctan *n*, where *n* is the refractive index of the reflecting material, and θ_B_ is measured from the surface’s normal vector). If β departs from θ_B_, *d* monotonously decreases and reaches 0 at β = 0^o^ and 90°. The direction (angle) of polarisation is always perpendicular to the plane of reflection. A polarisation-sensitive tabanid flying around a given tilted test surface perceives surface-reflected light with continuously changing degree and angle of linear polarisation as demonstrated in Figs. 1 and 2. The reflection-polarisation characteristics of the matte and glass-covered matte test surfaces used in experiment 2 were measured by Fritz et al. (2020b). Depending on the direction of sunlight, these test surfaces were similarly polarising, or the matte surface was much less polarising than the shiny one. The direction of polarisation of light reflected from the shiny (smooth) test surface was always horizontal, independent of the sun’s direction. The matte surface reflected horizontally polarised light only if the skylight came from the front.

**Fig. 3:**
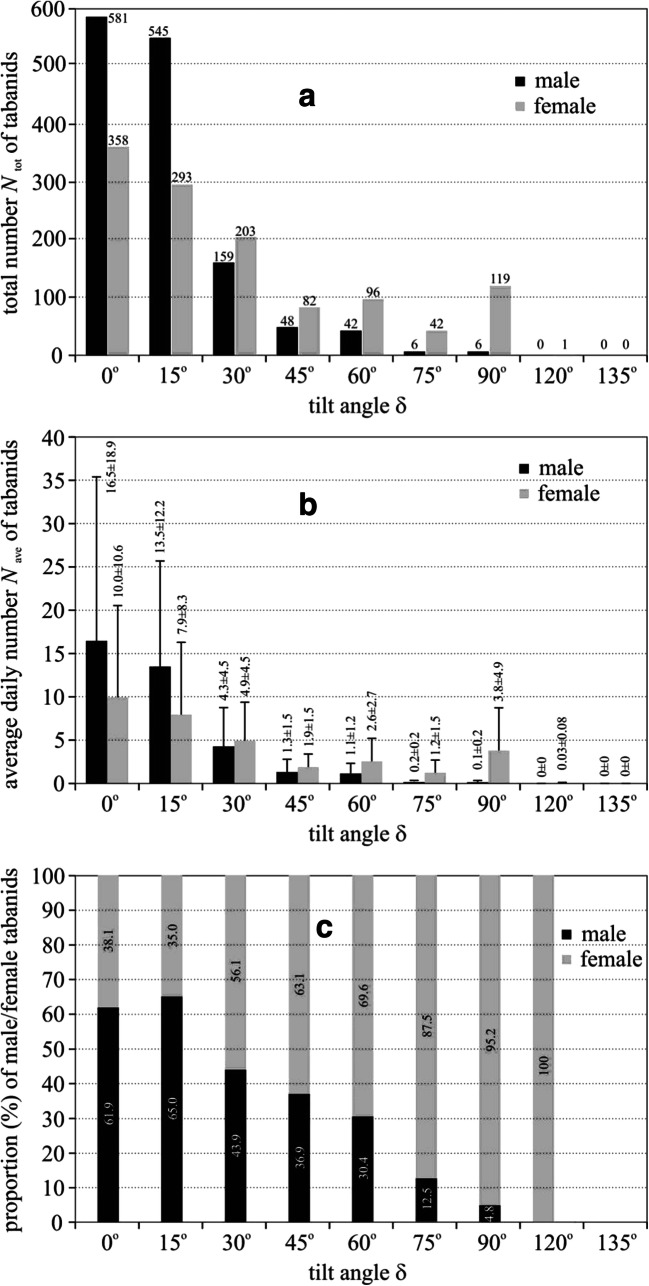
Total number *N*_tot_ (**a**), average daily number *N*_ave_ (**b**) and proportion % (**c**) of male (black) and female (grey) tabanid flies captured by the sticky tilted black test surfaces in experiment 1 as a function of the tilt angle δ (Supplementary Tables S1-S2). In **b** vertical bars denote standard deviations

The total number *N*_tot_ of male tabanids captured by the sticky tilted black test surfaces decreased monotonously from 581 to 6 with tilt angle δ increasing from 0 to 90° as seen in Fig. 3a and Supplementary Tables S1-S2. *N*_tot_ of captured females decreased tendentiously with increasing δ up to δ = 75° where it was minimal (42), while it reached a secondary maximum (119) at δ = 90°. For δ = 120° and 135°, the test surfaces practically did not trap tabanids.

For handling the non-uniform sampling periods of tabanids trapped by the test surfaces, before performing any statistical tests, the catch numbers between two consecutive samplings were normalized with the number of days passed since the previous sampling. For the first sampling, the installation of the experimental setup was taken into account. This normalization resulted in the average *N*_ave_ and standard deviation of daily catches of tabanids for the days between the sampling dates, for which statistical tests were performed. *N*_ave_ of males decreased monotonously from 16.46 ± 18.92 to 0.14 ± 0.20 with δ increasing from 0 to 90° as shown in Fig. 3b. *N*_ave_ of females decreased tendentiously with increasing δ up to δ = 75° where it was minimal (1.23 ± 1.46), while it reached a secondary maximum (3.79 ± 4.94) at δ = 90°. Using factorial ANOVA, considering the tilt angle δ, highly significant differences were found in *N*_ave_ (SS = 2247.164, df = 8, MS = 280.895, *F* = 6.58231, *p* < 0.0001). On the other hand, regarding the sex (male, female) of tabanids, no significant differences were found globally in *N*_ave_ (SS = 7.284, df = 1, MS = 7.284, *F* = 0.17068, *p* = 0.6805). According to the Tukey HSD test (df = 90), the male catch of the horizontal (δ = 0°) test surface was significantly different (*p* < 0.05) from that of both males and females on the surfaces with δ ≥ 45° except for one case. The female catch of the vertical (δ = 90°) surface was not significantly different (*p* = 0.948) from the male catch of the horizontal surface.

According to Fig. 3c, the proportion π_m_ of trapped males had a maximum (65%) at δ = 15° from where it monotonously decreased with increasing δ, reaching a minimum (0%) at δ = 120°. The change of the proportion π_f_(δ) of females versus δ was the inverse/complementary of π_m_(δ), because π_f_(δ) = 1 − π_m_(δ).

### Influence of surface temperature on tabanid behaviour (experiment 2)

**Fig. 4:**
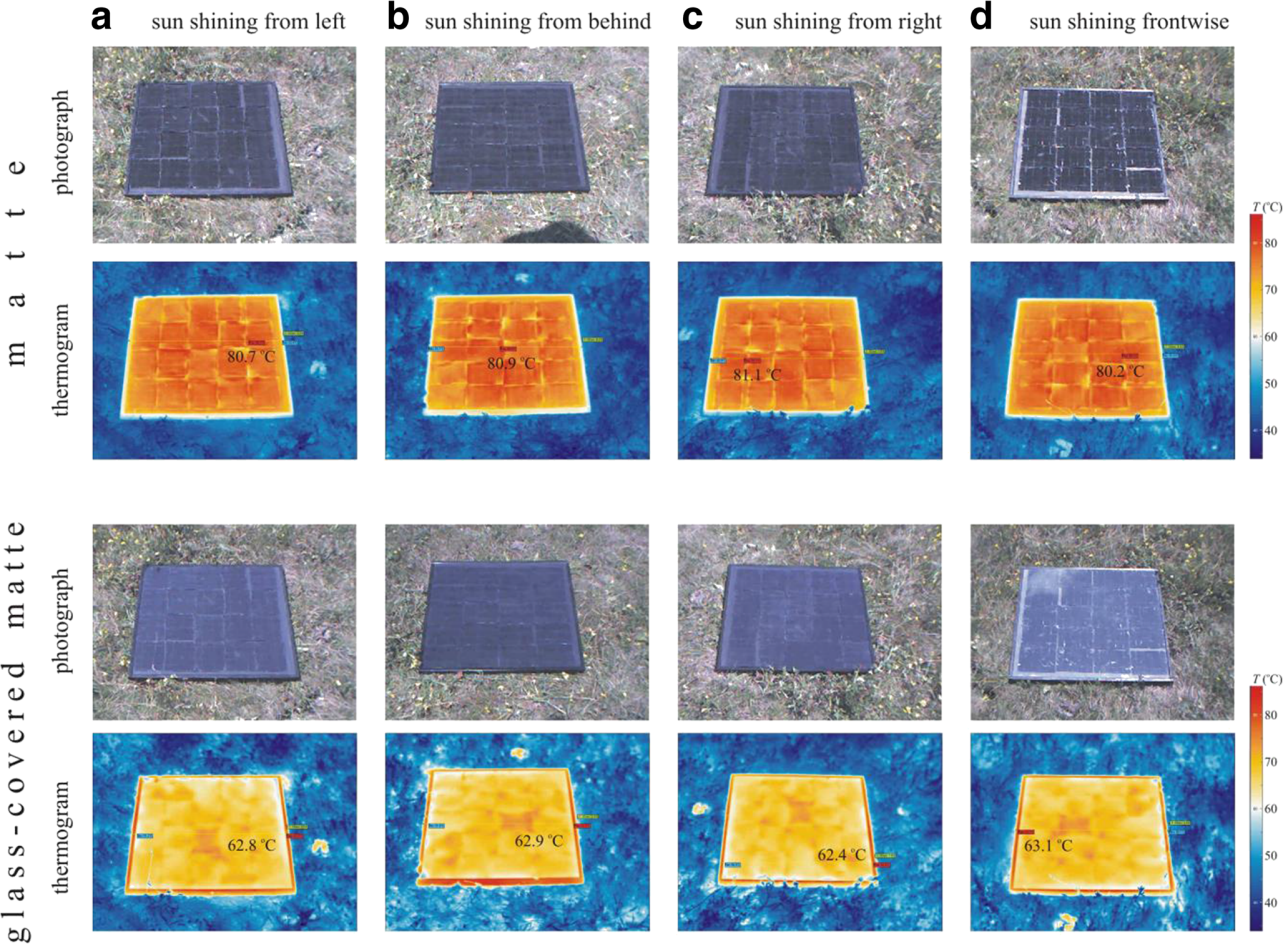
Photographs and thermograms of the sunlit horizontal matte black and glass-covered matte black test surfaces used in experiment 2 and measured by thermocamera from four different directions of view when the sun shone from left (**a**), behind (**b**), right (**c**) and front (**d**). The maximum temperatures of the test surfaces are given in the thermograms

Due to the slight curving of the matte surface, its temperature distribution was slightly inhomogeneous with ± 2 °C scatter as shown in Fig. 4. At the time of measurement with 35 °C air temperature, its average temperature reached up to 80.5 °C, independently of the sun’s direction. Due to the larger surface reflectivity and thus smaller absorbance of the smooth glass pane, the glass-covered matte surface was cooler with an average temperature of 62.6 °C. Regardless of the solar elevation, the matte surface was always warmer than its glass-covered counterpart (Supplementary Fig. S2) due to the higher absorption of the former which was designed to minimize light reflectance over a broad range of incidence angles.

**Fig. 5:**
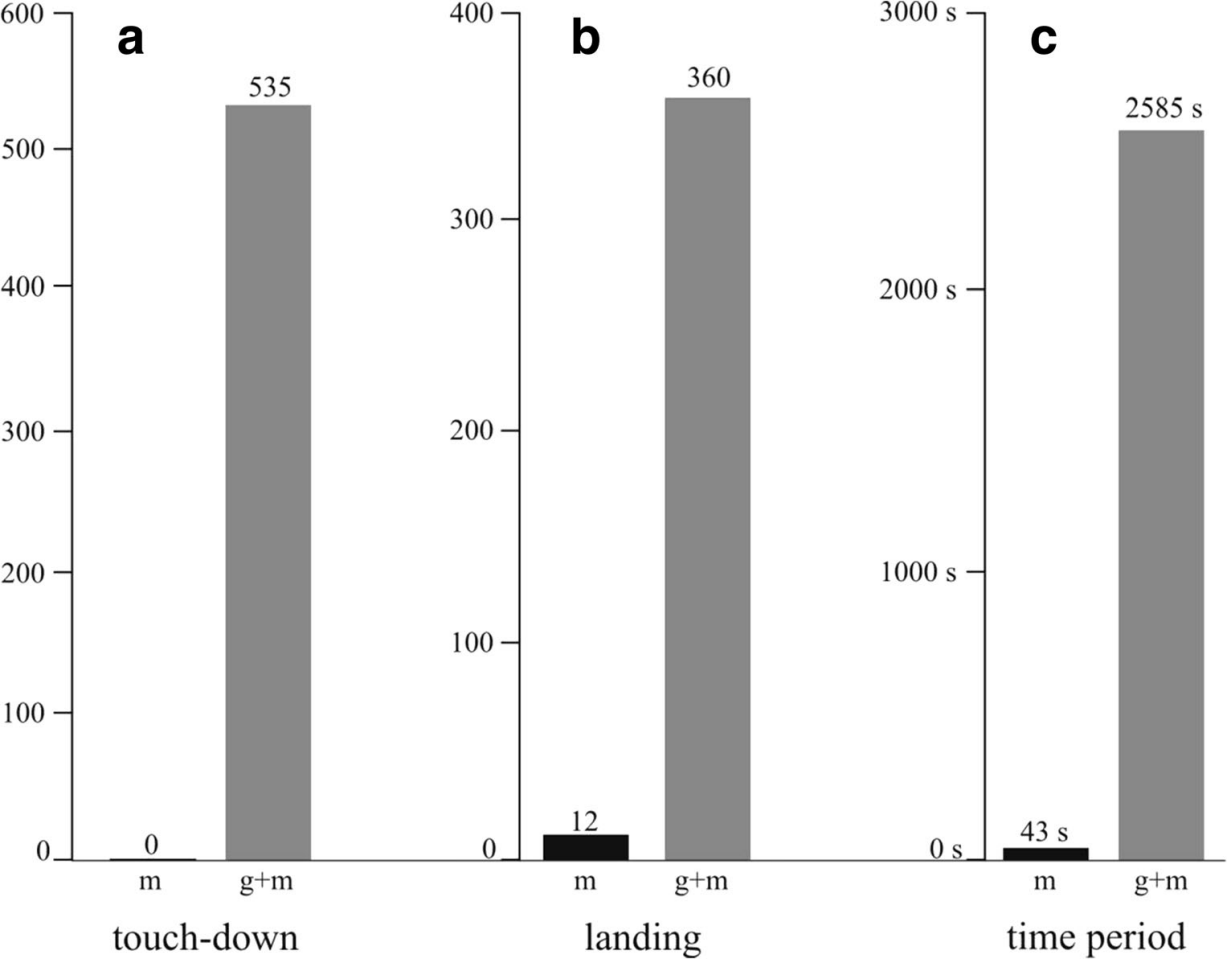
Number of reactions (touchdown, landing) and time periods (in seconds) spent by tabanid flies on the test surfaces (*m*, matte black, *g* + *m*, glass-covered matte black) in experiment 2

Considering the numbers of reactions (touchdowns, landings) of tabanid flies and their time periods spent on the matte and glass-covered matte horizontal test surfaces, the matte test surface was practically unattractive to tabanids as seen in Fig. 5.

**Fig. 6:**
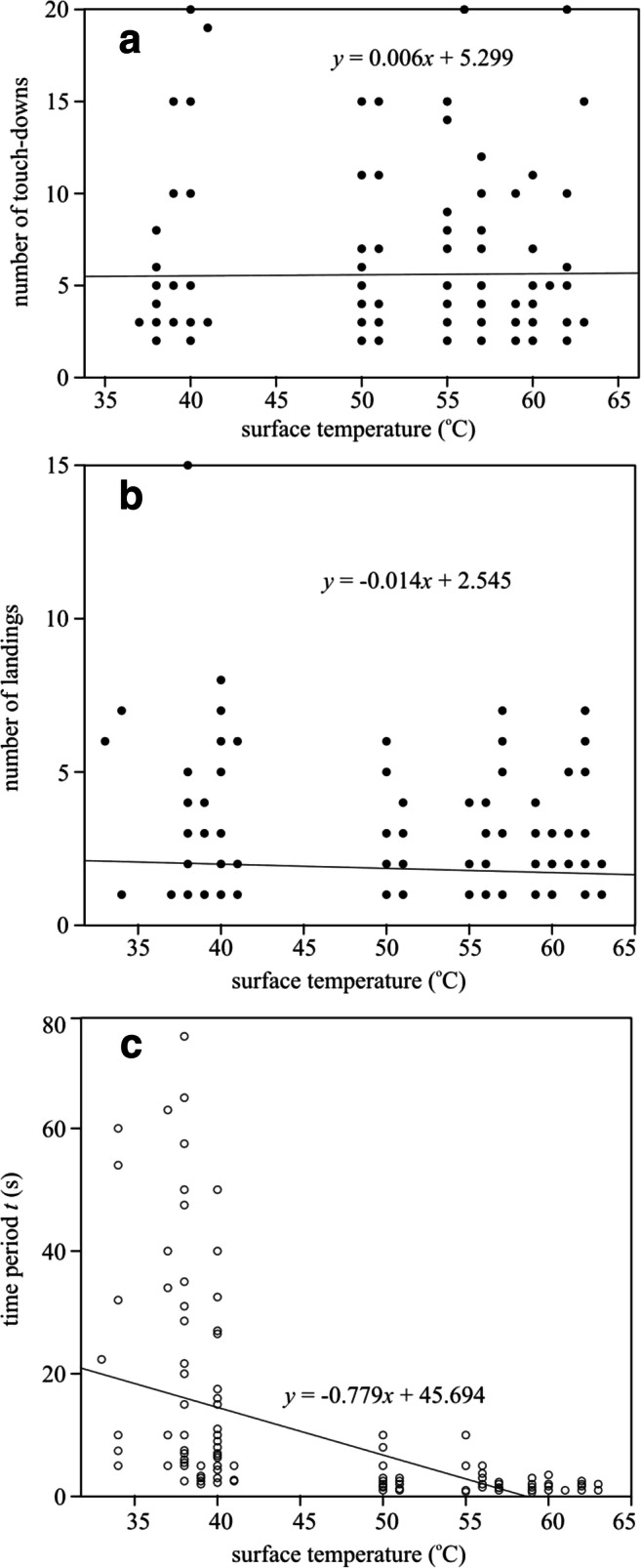
Number of touchdowns (**a**), number of landings (**b**) and time period spent on the glass-covered matte black test surface (**c**) of tabanid flies versus surface temperature (°C) measured in experiment 2 (Supplementary Table S3) and the regression line *y*(*x*) fitted to the data points

The number of touchdowns *N*_touch_ of tabanid flies was independent of the temperature *T* (measured with a contact thermometer, see Supplementary Table S3) as proven by the practically horizontal regression line in Fig. 6a. Although the number of landings *N*_land_ of tabanids slightly decreased with increasing surface *T*, it was practically independent of *T* as shown by the almost horizontal regression line in Fig. 6b (see also Supplementary Table S3). The time period *t* of tabanids spent on the test surface decreased with increasing surface temperature *T* so that above 58 °C tabanids spent not longer than 1 s on the surface as displayed in Fig. 6c (see also Supplementary Table S3).

## Discussion

The higher the degree of linear polarisation *d* of light reflected from a target, the larger is its attractiveness to tabanid flies (Kriska et al. 2009; Egri et al. 2013). At a given viewing direction, the *d* of light reflected from our tilted test surfaces depended on the tilt angle δ (Figs. 1 and 2). One could imagine that the different attractiveness to tabanids of our oblique test surfaces could be partly explained with the dependence of *d* on δ. However, this explanation would be false, because prior to landing, a horsefly circles around the target and perceives continuously changing *d* of target-reflected light due to the continuously altering viewing direction. As a matter of fact, *d* depends only on the angle of reflection β from the normal vector of the reflecting surface (see inset in the top right corner of Fig. 1). The perceived time-averaged *d* of light reflected from our nine tilted test surfaces was the same for circling tabanids. Thus, the measured different tabanid attractiveness of our tilted test surfaces was not due to the different *d* values sensed from a given viewing direction.

Since the amount of sunlight absorbed by a surface depends on the angle of incidence β from the normal vector of the reflecting surface (see inset in the top right corner of Fig. 1), the temperature *T* of our tilted test surfaces was different in direct sunshine: the smaller the β, the warmer the surface, the temperature of which is maximal for β = 0°. One could assume that the different attractiveness to tabanids of our sunlit oblique test surfaces used in experiment 1 could be partly explained with their different surface temperatures. However, tabanid flies do not have infrared-sensitive receptors with which they could perceive remotely the temperature of a target in flight, thus they can sense the surface temperature only after landing on the target. We found in experiment 2 that the number of touchdowns (Fig. 6a) and landings (Fig. 6b) is independent of the surface temperature. Therefore, the different tabanid attractiveness of the tilted test surfaces observed in experiment 1 was not due to the different surface temperatures, because the sticky adhesive captured all horseflies which touched it.

The reflection-polarisation characteristics of our test surfaces with tilt angle δ = 120° and 135° were not measured, because they were almost always shady, and thus, too dark for polarimetry. However, depending on the tilt angle and direction of view, their degree and angle of linear polarisation were similar to those of the other tilted test surfaces. In spite of this, they were unattractive to horseflies.

From the results of our field experiment 1, we conclude the following: The total number of trapped (male and female) tabanids is highest if the surface is horizontal (δ = 0°), and it is minimal at δ = 75°. The number of trapped males decreases monotonously to zero with increasing δ, while the female catch has a primary maximum and minimum at δ = 0° and δ = 75°, respectively, furthermore a secondary peak at δ = 90°. Both sexes are strongly attracted to nearly horizontal (0° ≤ δ ≤ 15°) surfaces, and the vertical surface is also very attractive, but only for females. Much less tabanid flies landed on the sunlit matte black test surface than on the shiny black surfaces, because (i) the matte surface was much less polarising, (ii) the matte-reflected light was not always horizontal and (iii) the matte surface was warmer than the glass-covered matte surface.

Tabanids can sense precisely the temperature of a substrate only after landing. Our field experiment showed that the numbers of touchdowns and landings do not depend on the temperature of the horizontal test surfaces (Fig. 6a, b), because tabanids could feel this temperature only after their alighting. However, the time spent on the test surface is highly dependent on the surface temperature (Fig. 6c): the higher this temperature, the shorter period is spent by tabanids on the surface. Above 58 °C, tabanids do not spend more time than about 1 s on a surface such as a solar panel. Hence, tabanid flies are not happy to walk around on very warm surfaces. These results suggest that the attractiveness of solar panels do not depend on the surface temperature, but if the surface of the solar panel is hot (above 58 °C, which can be easily reached during operation), the horseflies do not stay. This effect may reduce the negative ecological impact of solar panels on polarotactic insect species.

From the results presented here, it follows that the optimal tilt angle of a sticky black planar horsefly trap is δ = 0°, when the trap is laying horizontal on the ground. In this case, the trap captures maximal numbers of male and female tabanids which look for water. The second optimal tilt angle is δ = 90°, when the vertical trap captures efficiently only host-seeking tabanids. The worst tilt angle is δ = 75°, when the trap captures minimal numbers of horseflies. Our results obtained for δ = 0° and 90° are consistent with the earlier results of Horváth et al. (2014b). In case of a sticky black planar horsefly trap, the attractiveness does not depend on the surface temperature, because the sticky adhesive captures the fly at its touchdown.

In experiment 2, we found that the matte test surface was unattractive while its glass-covered counterpart was attractive to tabanids (Fig. 5). This result can be used to reduce the polarised light pollution of solar panels (Horváth et al. 2010b; Blahó et al. 2012a; Száz et al. 2016). The horizontally polarised light reflected from solar panels attracts aquatic insects and insects associated with water, because they detect water by means of the horizontal polarisation of water-reflected light. This attraction is adverse, if the lured insects lay their eggs onto the black surface and/or cannot escape from the polarised signal and perish due to dehydration. If solar panels are provided with the matte cover layer used in this work, their polarised light pollution (being proportional to the visual attractiveness to polarotactic insects) can be minimized or eliminated.

Beyond tabanid flies, it would be worth performing analogous field experiments also with other polarotactic insect species that are also victims of the polarised light pollution caused by solar panels (Horváth et al. 2010b; Blahó et al. 2012a; Száz et al. 2016). These insects may react differently to tilt angle and surface temperature.

## Electronic supplementary material

ESM 1 (DOC 1120 kb)

## Data Availability

Our paper has the following electronic supporting material: Supplementary Tables S1, S2, S3 and Supplementary Figures S1, S2.
