# Supplementary material for: Horsefly reactions to black surfaces: attractiveness to male and female tabanids versus surface tilt angle and temperature
Source: Parasitol Res. 2020 May 19;119(8):2399–409. doi: 10.1007/s00436-020-06702-7 (PMC7366589; doi:10.1007/s00436-020-06702-7)
Supplement: Supplementary file 1 — (DOC 1120 kb) [file 436_2020_6702_MOESM1_ESM.doc]

**Electronic Supporting Material**

for

**Horsefly reactions to black surfaces: Attractiveness to male and female tabanids versus surface tilt angle and temperature**

Gábor Horváth*, Ádám Pereszlényi, Ádám Egri, Benjamin Fritz, Markus Guttmann,

Uli Lemmer, Guillaume Gomard and György Kriska

*: correspondig author: Environmental Optics Laboratory, Department of Biological Physics,

ELTE Eötvös Loránd University, H-1117 Budapest, Pázmány sétány 1, Hungary,

e-mail: gh@arago.elte.hu

This file contains the following: Supplementary Tables S1, S2, S3

Supplementary Figures S1, S2

**Supplementary Table S1**: Number and percentage (%) of male and female tabanid flies trapped by the sticky test surfaces with different tilt angles δ measured from the horizontal in experiment 1. m: male, f: female.

| **date**  **(2019)** | **tilt angle δ of the test surface from the horizontal** | | | | | | | | |
| --- | --- | --- | --- | --- | --- | --- | --- | --- | --- |
|  | **0^o^** | **15^o^** | **30^o^** | **45^o^** | **60^o^** | **75^o^** | **90^o^** | **120^o^** | **135^o^** |
| 4 July | 199 m  (66.33%)  101 f  (33.67%) | 93 m  (51.67%)  87 f  (48.33%) | 38 m  (43.18%)  50 f  (56.82%) | 9 m  (42.86%)  12 f  (57.14%) | 10 m  (33.33%)  20 f  (66.67%) | 2 m  (14.29%)  12 f  (85.71%) | 2 m  (4.35%)  44 f  (95.65%) | 0 m  (0%)  0 f  (0%) | 0 m  (0%)  0 f  (0%) |
| 9 July | 143 m  (56.97%)  108 f  (43.03%) | 166 m  (69.46%)  73 f  (30.54%) | 52 m  (57.14%)  39 f  (42.86%) | 19 m  (47.5%)  21 f  (52.5%) | 13 m  (28.26%)  33 f  (71.74%) | 1 m  (5.88%)  16 f  (94.12%) | 0 m  (0%)  46 f  (100%) | 0 m  (0%)  1 f  (100%) | 0 m  (0%)  0 f  (0%) |
| 19 July | 66 m  (57.39%)  49 f  (42.61%) | 110 m  (75.86%)  35 f  (24.14%) | 19 m  (47.5%)  21 f  (52.5%) | 12 m  (40%)  18 f  (60%) | 13 m  (43.33%)  17 f  (56.67%) | 1 m  (20%)  4 f  (80%) | 1 m  (7.69%)  12 f  (92.31%) | 0 m  (0%)  0 f  (0%) | 0 m  (0%)  0 f  (0%) |
| 1 August | 65 m  (57.52%)  48 f  (42.48%) | 87 m  (60.42%)  57 f  (39.58%) | 35 m  (38.46%)  56 f  (61.54%) | 5 m  (19.23%)  21 f  (80.77%) | 1 m  (4.35%)  22 f  (95.65%) | 2 m  (20%)  8 f  (80%) | 3 m  (18.75%)  13 f  (81.25%) | 0 m  (0%)  0 f  (0%) | 0 m  (0%)  0 f  (0%) |
| 12 August | 80 m  (67.79%)  38 f  (32.21%) | 52 m  (61.90%)  32 f  (38.10%) | 8 m  (26.67%)  22 f  (73.33%) | 3 m  (42.86%)  4 f  (57.14%) | 3 m  (60%)  2 f  (40%) | 0 m  (0%)  2 f  (100%) | 0 m  (0%)  4 f  (100%) | 0 m  (0%)  0 f  (0%) | 0 m  (0%)  0 f  (0%) |
| 30 August | 28 m  (66.67%)  14 f  (33.33%) | 37 m  (80.43%)  9 f  (19.57%) | 7 m  (31.82%)  15 f  (68.18%) | 0 m  (0%)  6 f  (100%) | 2 m  (50%)  2 f  (50%) | 0 m  (0%)  0 f  (0%) | 0 m  (0%)  0 f  (0%) | 0 m  (0%)  0 f  (0%) | 0 m  (0%)  0 f  (0%) |
| **sum** | **581 m**  **(61.9%)**  **358 f**  **(38.1%)** | **545 m**  **(65.0%)**  **293 f**  **(35.0%)** | **159 m**  **(43.9%)**  **203 f**  **(56.1%)** | **48 m**  **(36.9%)**  **82 f**  **(63.1%)** | **42 m**  **(30.4%)**  **96 f**  **(69.6%)** | **6 m**  **(12.5%)**  **42 f**  **(87.5%)** | **6 m**  **(4.8%)**  **119 f**  **(95.2%)** | **0 m**  **(0%)**  **1 f**  **(100%)** | **0 m**  **0 f** |

**Supplementary Table S2**: Average daily number *N*_ave_ ± standard deviation of male and female tabanids captured by the sticky tilted black test surfaces in experiment 1 as a function of the tilt angle δ.

| **δ** | **0^o^** | **15^o^** | **30^o^** | **45^o^** | **60^o^** | **75^o^** | **90^o^** | **120^o^** | **135^o^** |
| --- | --- | --- | --- | --- | --- | --- | --- | --- | --- |
| male | 16.5±18.9 | 13.5±12.2 | 4.3±4.5 | 1.3±1.5 | 1.1±1.2 | 0.2±0.2 | 0.1±0.2 | 0±0 | 0±0 |
| female | 10.0±10.6 | 7.9±8.3 | 4.9±4.5 | 1.9±1.5 | 2.6±2.7 | 1.2±1.5 | 3.8±4.9 | 0.03±0.08 | 0±0 |

**Supplementary Table S3**: Numbers of reactions (T: number of touch-down, L: number of landing, s: seconds spent on the surface) of tabanid flies and temperature (^o^C, measured with a contact thermometer) for the matte and glass-covered matt horizontal black test surfaces used in experiment 2 with start and end times (UTC + 2 hours) in brackets.

| **date in 2019**  **(UTC + 2 h)** | **matte black** | **glass-covered matte black** |
| --- | --- | --- |
| 18 June  (9:30-13:00) | 0T, 1L, 3s, 39^o^C  0T, 1L, 2s, 39^o^C  0T, 1L, 5s, 41^o^C  0T, 1L, 2s, 56^o^C  0T, 1L, 1s, 59^o^C  **SUM: 0T, 5L, 13s** | 0T, 1L, 10s, 37^o^C  0T, 1L, 63s, 37^o^C  3T, 1L, 34s, 37^o^C  0T, 1L, 5s, 38^o^C  3T, 1L, 10s, 38^o^C  3T, 1L, 5s, 38^o^C  5T, 2L, 15s, 38^o^C  3T, 2L, 5s, 50^o^C  0T, 1L, 3s, 50^o^C  2T, 2L, 5s, 50^o^C  0T, 3L, 5s, 50^o^C  0T, 3L, 6s, 50^o^C  0T, 2L, 5s, 50^o^C  0T, 1L, 3s, 50^o^C  0T, 2L, 5s, 50^o^C  0T, 4L, 15s, 56^o^C  0T, 2L, 4s, 56^o^C  0T, 2L, 6s, 56^o^C  0T, 6L, 12s, 57^o^C  0T, 5L, 7s, 57^o^C  12T, 1L, 1s, 57^o^C  5T, 7L, 10s, 57^o^C  0T, 5L, 5s, 57^o^C  4T, 3L, 5s, 60^o^C  3T, 1L, 2s, 60^o^C  **SUM: 43T, 60L, 246s** |
| 19 June  (9:00-12:00) | 0T, 1L, 5s, 34^o^C  **SUM: 0T, 1L, 5s** | 0T, 2L, 37s, 33^o^C  0T, 6L, 134s, 33^o^C  0T, 1L, 54s, 34^o^C  0T, 1L, 32s, 34^o^C  0T, 7L, 52s, 34^o^C  **SUM: 0T, 17L, 309s** |
| 25 June  (9:45-14:00) | 0T, 1L, 2s, 56^o^C  **SUM: 0T, 1L, 2s** | 0T, 1L, 8s, 40^o^C  4T, 3L, 7s, 57^o^C  3T, 2L, 7s, 60^o^C  0T, 2L, 5s, 62^o^C  0T, 1L, 2s, 62^o^C  20T, 3L, 5s, 62^o^C  **SUM: 27T, 12L, 34s** |
| 27 June  (9:50-14:00) | 0T, 0L, 0s  **SUM: 0T, 0L, 0s** | 0T, 1L, 20s, 38^o^C  0T, 1L, 50s, 38^o^C  0T, 1L, 15s, 38^o^C  3T, 4L, 22s, 38^o^C  0T, 1L, 7s, 38^o^C  0T, 1L, 5s, 55^o^C  0T, 1L, 10s, 55^o^C  3T, 4L, 3s, 55^o^C  2T, 1L, 1s, 57^o^C  0T, 1L, 1s, 60^o^C  0T, 1L, 2s, 62^o^C  0T, 2L, 2s, 62^o^C  0T, 3L, 3s, 62^o^C  5T, 7L, 7s, 62^o^C  5T, 5L, 5s, 62^o^C  **SUM: 18T, 34L, 153s** |
| 6 July  (9:45-16:00) | 0T, 1L, 15s, 38^o^C  0T, 1L, 5s, 41^o^C  0T, 1L, 1s, 51^o^C  0T, 2L, 2s, 59^o^C  **SUM: 0T, 5L, 23s** | 0T, 1L, 5s, 37^o^C  0T, 1L, 40s, 37^o^C  0T, 2L, 115s, 38^o^C  0T, 1L, 35s, 38^o^C  8T, 3L, 60s, 38^o^C  5T, 3L, 65s, 38^o^C  5T, 15L, 90s, 38^o^C  6T, 5L, 143s, 38^o^C  5T, 1L, 75s, 38^o^C  5T, 1L, 5s, 38^o^C  2T, 1L, 65s, 38^o^C  3T, 1L, 15s, 38^o^C  4T, 4L, 40s, 38^o^C  4T, 1L, 31s, 38^o^C  2T, 2L, 95s, 38^o^C  0T, 2L, 5s, 38^o^C  0T, 3L, 15s, 38^o^C  0T, 1L, 35s, 38^o^C  2T, 2L, 35s, 40^o^C  10T, 2L, 65s, 40^o^C  0T, 1L, 15s, 40^o^C  0T, 8L, 18s, 40^o^C  0T, 1L, 5s, 40^o^C  0T, 6L, 26s, 40^o^C  0T, 2L, 10s, 40^o^C  0T, 1L, 8s, 40^o^C  0T, 7L, 21s, 40^o^C  0T, 1L, 3s, 40^o^C  0T, 1L, 10s, 40^o^C  0T, 1L, 50s, 40^o^C  0T, 3L, 27s, 40^o^C  5T, 2L, 30s, 40^o^C  0T, 1L, 5s, 40^o^C  0T, 5L, 15s, 40^o^C  0T, 6L, 38s, 40^o^C  20T, 1L, 40s, 40^o^C  0T, 1L, 16s, 40^o^C  15T, 1L, 10s, 40^o^C  0T, 1L, 10s, 40^o^C  0T, 3L, 48s, 40^o^C  5T, 1L, 10s, 40^o^C  0T, 1L, 5s, 40^o^C  3T, 6L, 40s, 40^o^C  0T, 2L, 53s, 40^o^C  0T, 1L, 27s, 40^o^C  0T, 1L, 10s, 40^o^C  2T, 1L, 9s, 40^o^C  2T, 1L, 11s, 40^o^C  0T, 1L, 5s, 40^o^C  0T, 1L, 7s, 40^o^C  0T, 1L, 5s, 40^o^C  0T, 2L, 10s, 40^o^C  0T, 1L, 10s, 40^o^C  0T, 1L, 15s, 40^o^C  2T, 1L, 10s, 40^o^C  3T, 1L, 10s, 40^o^C  0T, 1L, 10s, 50^o^C  11T, 1L, 2s, 50^o^C  5T, 1L, 5s, 50^o^C  5T, 2L, 10s, 50^o^C  2T, 1L, 8s, 50^o^C  4T, 1L, 3s, 50^o^C  5T, 1L, 5s, 50^o^C  5T, 1L, 1s, 50^o^C  6T, 1L, 2s, 50^o^C  3T, 1L, 1s, 50^o^C  0T, 1L, 3s, 50^o^C  0T, 1L, 1s, 50^o^C  5T, 2L, 3s, 50^o^C  0T, 6L, 6s, 50^o^C  7T, 1L, 2s, 50^o^C  0T, 1L, 3s, 50^o^C  4T, 1L, 1s, 50^o^C  0T, 1L, 1s, 50^o^C  0T, 1L, 1s, 50^o^C  3T, 1L, 1s, 50^o^C  2T, 1L, 1s, 50^o^C  0T, 2L, 2s, 50^o^C  15T, 5L, 5s, 50^o^C  2T, 1L, 2s, 50^o^C  3T, 1L, 1s, 55^o^C  15T, 1L, 1s, 55^o^C  8T, 1L, 2s, 55^o^C  0T, 1L, 1s, 55^o^C  2T, 1L, 1s, 55^o^C  3T, 1L, 1s, 55^o^C  9T, 1L, 1s, 55^o^C  3T, 1L, 1s, 55^o^C  7T, 1L, 1s, 55^o^C  3T, 1L, 1s, 55^o^C  3T, 1L, 1s, 55^o^C  8T, 1L, 1s, 55^o^C  7T, 2L, 2s, 55^o^C  14T, 1L, 1s, 55^o^C  8T, 1L, 1s, 55^o^C  3T, 1L, 1s, 55^o^C  5T, 1L, 1s, 55^o^C  3T, 1L, 1s, 55^o^C  3T, 1L, 1s, 55^o^C  2T, 1L, 1s, 55^o^C  0T, 1L, 1s, 55^o^C  2T, 1L, 1s, 55^o^C  4T, 2L, 2s, 55^o^C  0T, 1L, 1s, 57^o^C  3T, 1L, 1s, 57^o^C  3T, 1L, 1s, 57^o^C  7T, 1L, 2s, 57^o^C  0T, 1L, 1s, 57^o^C  0T, 1L, 1s, 57^o^C  8T, 1L, 1s, 57^o^C  3T, 1L, 1s, 57^o^C  10T, 1L, 1s, 57^o^C  3T, 1L, 1s, 57^o^C  2T, 1L, 1s, 57^o^C  10T, 1L, 1s, 57^o^C  5T, 1L, 1s, 57^o^C  2T, 1L, 2s, 60^o^C  0T, 1L, 1s, 60^o^C  0T, 1L, 1s, 60^o^C  3T, 1L, 1s, 62^o^C  2T, 1L, 1s, 62^o^C  2T, 1L, 1s, 62^o^C  5T, 2L, 2s, 62^o^C  0T, 1L, 1s, 59^o^C  2T, 1L, 1s, 59^o^C  0T, 2L, 2s, 59^o^C  0T, 1L, 1s, 59^o^C  0T, 2L, 2s, 59^o^C  10T, 3L, 2s, 59^o^C  3T, 1L, 1s, 60^o^C  5T, 1L, 1s, 60^o^C  0T, 1L, 1s, 60^o^C  7T, 1L, 1s, 60^o^C  0T, 1L, 2s, 60^o^C  0T, 1L, 1s, 60^o^C  0T, 1L, 1s, 60^o^C  11T, 1L, 1s, 60^o^C  11T, 1L, 2s, 51^o^C  2T, 0L, 0s, 51^o^C  4T, 0L, 0s, 51^o^C  3T, 0L, 0s, 51^o^C  0T, 3L, 3s, 51^o^C  0T, 1L, 3s, 51^o^C  0T, 3L, 3s, 50^o^C  2T, 0L, 0s, 50^o^C  2T, 1L, 10s, 50^o^C  **SUM: 447T, 237L, 1843s** |
| **TOTAL** | **0T, 12L, 43s** | **535T, 360L, 2585s** |


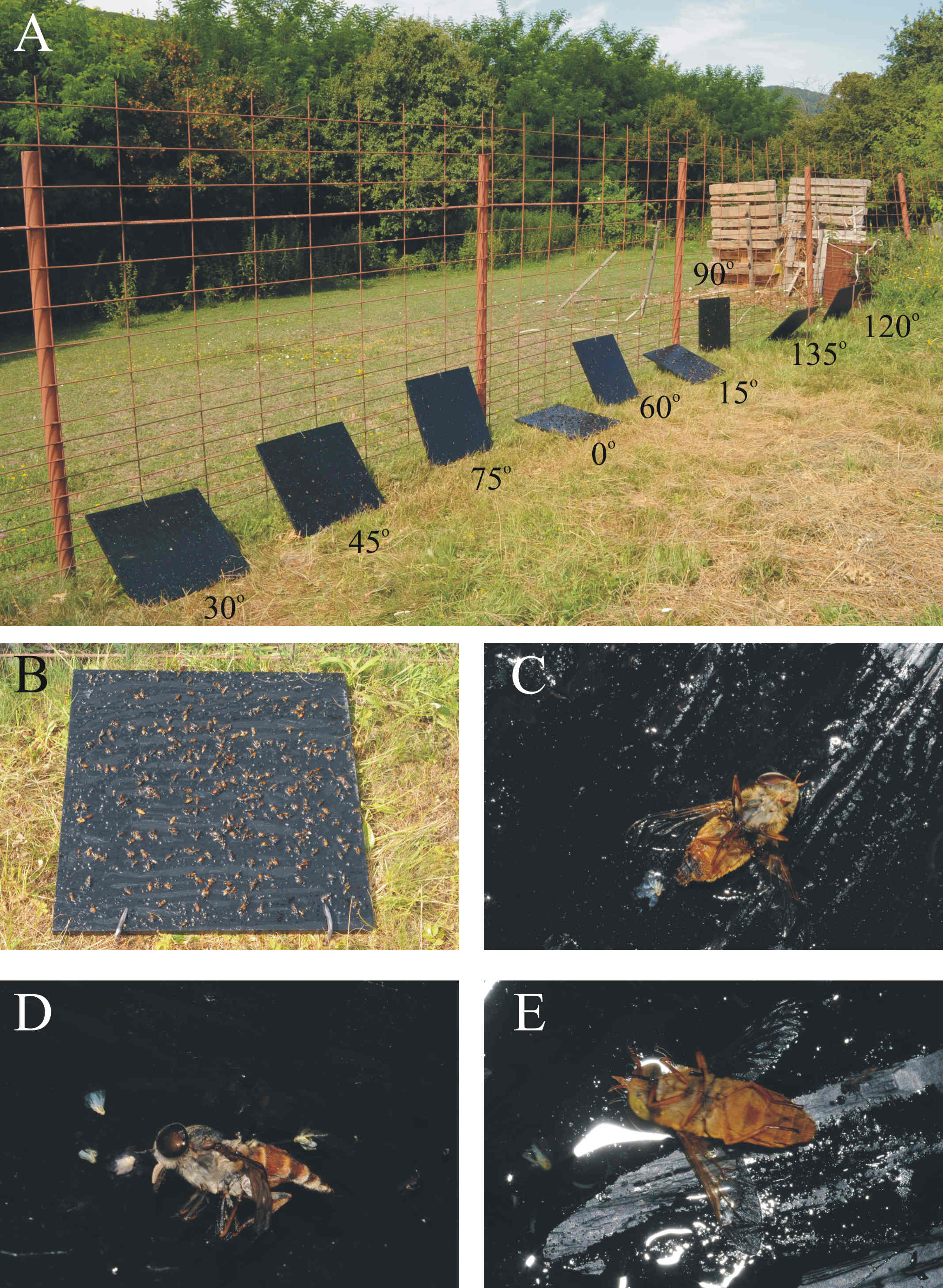


**Supplementary Figure S1:** Photographs of an arrangement of the tilted shiny black sticky test surfaces in experiment 1 (A), numerous tabanid flies trapped by the horizontal test surface (B), and examples for horseflies captured by the adhesive (C, D, E). In picture A, the tilt angles δ of the test surfaces are given.


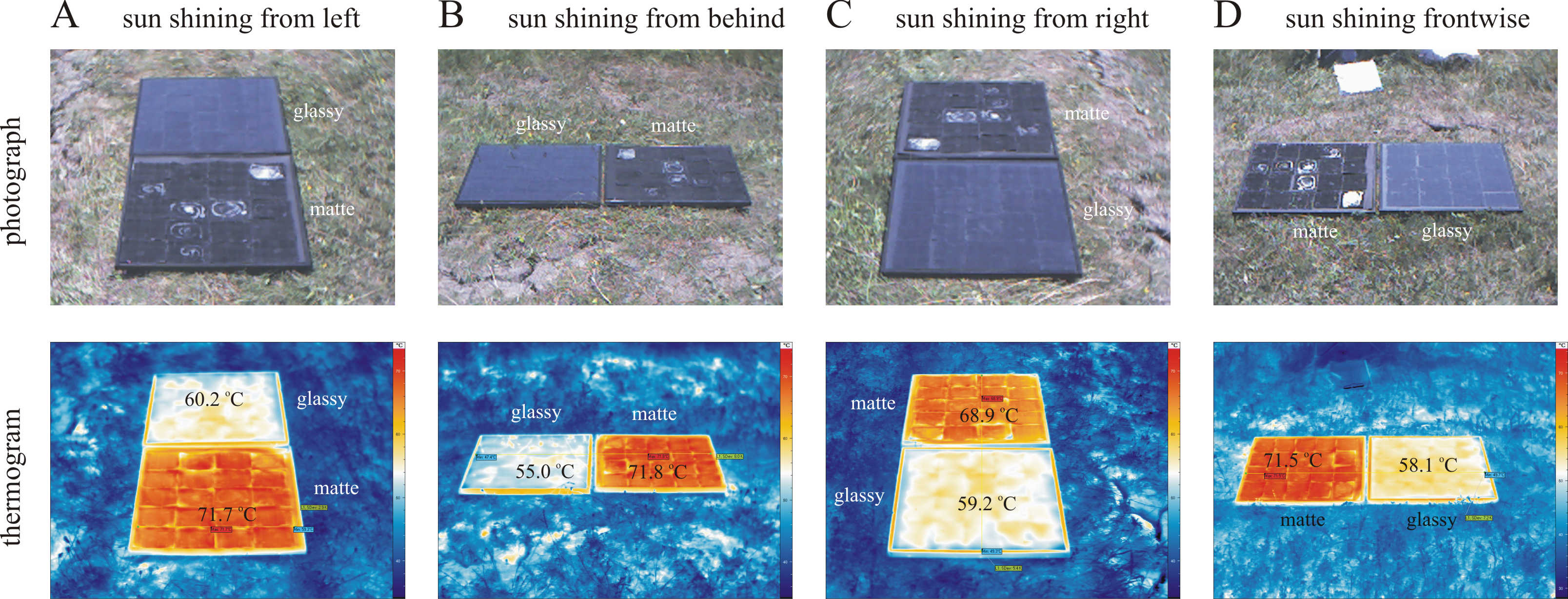


**Supplementary Figure S2:** Photographs and thermograms of the sunlit horizontal matte black and glass-covered matte black test surfaces used in experiment 2 and measured by thermocamera from four different directions of view when the sun shone from left (A), behind (B), right (C) and frontwise (D). The maximum temperatures of the test surfaces are given in the thermograms.
